# Congenital heart disease in neonates with external congenital anomalies in Jos, Nigeria

**DOI:** 10.4103/jomt.jomt_22_19

**Published:** 2020

**Authors:** OO IGE, CS YILGWAN, AS SAGAY, P KANKI, F BODE-THOMAS

**Affiliations:** 1 Department of Paediatrics, University of Jos/Jos University Teaching Hospital, Jos, Nigeria; 2 Department of Obstetrics and Gynaecology, University of Jos/Jos University Teaching Hospital, Jos, Nigeria; 3 Harvard TH Chan School of Public Health, Boston, USA

**Keywords:** Congenital heart defects, external congenital anomalies, neonates, Nigeria

## Abstract

**Background:**

Congenital heart disease (CHD) has been found to be more common in neonates with other congenital anomalies and may worsen prognosis. Early diagnosis and treatment of internal congenital anomalies including CHD will improve outcome and decrease neonatal mortality. This study determined the prevalence of CHD among neonates seen with external congenital anomalies in Jos, Nigeria.

**Materials and Methods:**

We performed complete physical examinations on 2,340 neonates delivered in two tertiary hospitals in Jos, Nigeria. We identified neonates with external congenital anomalies and determined the prevalence of congenital heart defects in them using echocardiography. Data were analysed using STATA 14.0.

**Results:**

External congenital anomalies were present in 49 of the 2,340 neonates recruited − prevalence of 20.9 per 1,000, with a male to female ratio of 1.1:1. Fourteen (28.6%) neonates were syndromic. CHD was present in 15 of 49 (30.6%) neonates studied– prevalence 30.6 per 100. The mean age of the parents with neonates who had external congenital anomalies and CHD was significantly higher than those without CHD.

**Conclusion:**

CHD frequently co-exists with external congenital anomalies especially in syndromic neonates. Hospital-based surveillance systems are needed to capture accurately both internal and external congenital anomalies to improve outcome in these group of neonates.

## INTRODUCTION

5Congenital anomalies (CA) refer to structural or functional disorders present in the fetus that can be identified prenatally, at birth or later in life.^[1]^ These anomalies have been found in about 20–40 babies per 1,000 live births and account for 17–42% of infant mortality.^[2]^ Since 2010, the proportion of neonatal deaths from CA worldwide has increased as a result of a concomitant decrease in mortality from infectious diseases.^[3]^

With over 300,000 annual deaths occurring worldwide from CA, stakeholders at the 63^rd^ World Health Assembly on CA resolved to improve the outcomes for these children by developing surveillance systems.^[1]^ Robust surveillance systems are however lacking in low- and middle-income countries like Nigeria especially for internal CA which are best detected in utero, delayed until days, weeks or months after delivery.^[4]^ While external CA are easily identified since they occur on visible parts, most internal CA require additional investigations such as X-rays, ultrasonography, computerized tomography (CT) scan and magnetic resonance imaging (MRI) to confirm diagnosis.^[5]^ However, these investigations are usually expensive for majority of those who suffer from this condition because medical care is still mainly out of pocket in Nigeria.^[6]^ The result of this is parents leaving the hospital with their children against medical advice without confirming the diagnosis.^[7]^

External CAs has been reported to co-exist with anomalies of internal organs.^[8]^ Congenital heart defects (CHD) have also been reported to be the most common CA occurring in up to 40% of cases.^[9]^ Also, the presence of CHD with other CA may worsen the clinical outcomes in affected neonates.^[10,11]^ Although recently, there have been a number of studies on the prevalence of CHD in neonates and older children with CA, there are no known reports conducted exclusively at birth in Nigeria.^[12,13]^

We therefore sought to determine the prevalence of CHD in neonates with external CA. This would strengthen advocacy for better hospital surveillance systems to estimate the current burden of CA and find ways to improve the outcome.

## METHODOLGY

### Study setting

This study was conducted in two tertiary hospitals in Jos, a city in north central Nigeria. Babies delivered in these hospitals were enrolled into the study over a period of two years from February 2017 to January 2019.

### Study design

This cross-sectional study is part of a larger study on the prevalence of CHD among neonates in Jos, Nigeria.

### Sampling technique

All babies delivered in these hospitals on weekdays were recruited between the hours of 8 am and 2 pm before discharge. Neonates delivered spend about 24 hours on admission after a normal delivery and five days after a caesarian section. It is expected that only a few babies born in these hospitals would have been missed if discharged too early or die within minutes of delivery.

### Data collection

Upon obtaining written consent from the primary caregiver, demographic and clinical data of the parents and neonates were obtained using a pre-tested proforma. Demographic information obtained include the age of mother, educational status and occupation of parents, gestational age of neonates as well as mode of delivery. Neonates were examined to determine the presence of external CA. Other data obtained include the weight and length of the neonates. Information collected was documented by trained research assistants. External CA were coded and classified using the International Classification of Disease version 10 (ICD 10) and European Surveillance of Congenital Anomalies (EUROCAT) malformation coding guide respectively.^[14,15]^ The anomalies were classified into syndromes and other CA which include isolated malformations, associations or sequences as defined below^[15]^:

1. Isolated malformations: A single developmental anomaly
2. Syndromes: A recognizable pattern of anomalies believed to be causally related
3. Associations: A pattern of anomalies, at least two of which are morphologic, with no identified causal relationship
4. Sequences: A single developmental anomaly which leads to a chain of secondary anomalies that may then lead to tertiary defects

In the absence of genetic testing for a more specific diagnosis, clinical diagnoses of syndromes, chromosomal and non-chromosomal, was made utilizing the National Health Institute (NIH) genetics home reference.^[16]^

Echocardiography was performed on all neonates recruited into the study with a portable General Electric (GE); Jiangsu, China) Vivid e machine (#5433170), using standard techniques and the type of CHD classified according to the ICD- 10 classification.^[14]^ Other congenital heart diseases such as left ventricular (LV) systolic dysfunction, hypertrophic cardiomyopathy (HOCM) and dextrocardia with no structural heart defect were also noted.

Data were analyzed using STATA 14.0 (Stata, College Station, Texas). The birth prevalence of external CA was determined. Frequency tables were used to display demographic and clinical parameters as well as pattern of CA detected with their associated CHD. T-test was used to test for associations between the presence of CHD and external congenital CA for numerical variables such as age of parents and anthropometric measurements of the neonates. Chisquare was used to test for association between categorical variables such as gender.

### Ethical approval

Approval was obtained from the Jos University Teaching Hospital Ethics Review Committee before the commencement of the study. Permission was also obtained from the hospitals and immunization center where the study was carried out. Written informed consent was obtained from the primary care giver before recruitment.

## RESULTS

External CA were detected in 49 of the 2,340 neonates delivered, yielding a prevalence of 20.9 per 1,000. However, if polydactyly, a minor congenital anomaly is excluded, the prevalence would be 13.2 per 1,000. Twenty-seven of the 49 (54.0%) neonates with external CA were male.

Table 1 shows the descriptive characteristics of neonates with CA and their parents. These neonates had a mean gestational age of 38.4±1.8 weeks and a mean birth weight of 3.1±0.7 kilograms − range of 1.4–4.8 kilograms. The mean length and occiptofrontal circumference were 48.5 ± 3.7cm and 34.7 ± 2.0 respectively. The mean age of the mothers of neonates with CA was 29.8 ± 6.8 years and 37.4 ± 7.1 years for the fathers. There was a history of previous child with CA in one neonate and 17/49 neonates had mothers with a previous history of miscarriage. Maternal HIV, hypertension and diabetes mellitus (DM) were present in 2/49 (4.1%), 5/49 (10.2%) and 1/49 (2.0%) cases respectively. None of the neonates in this study was a product of a consanguineous marriage.

The pattern of CA detected is shown in Table 2. Identifiable clinical syndromes accounted for 28.6% (14/49) neonates with external CA. Down syndrome was the most common syndrome and was identified in 78.7% (11/14) of them; prevalence of 4.7 per 1,000. Trisomy 18, Turner’s syndrome and Baraister-Winter syndrome were detected in one neonate each.

Non-syndromic CA detected were isolated anomalies and included Polydactyly 51.4%; anorectal malformation in 20.0% and omphalocoeles in 8.0%. Other anomalies detected included encepaholocoele and spina bifida in two (5.7%) neonates each and prune Belly syndrome in one neonate (2.9%).

**Figure 1:**
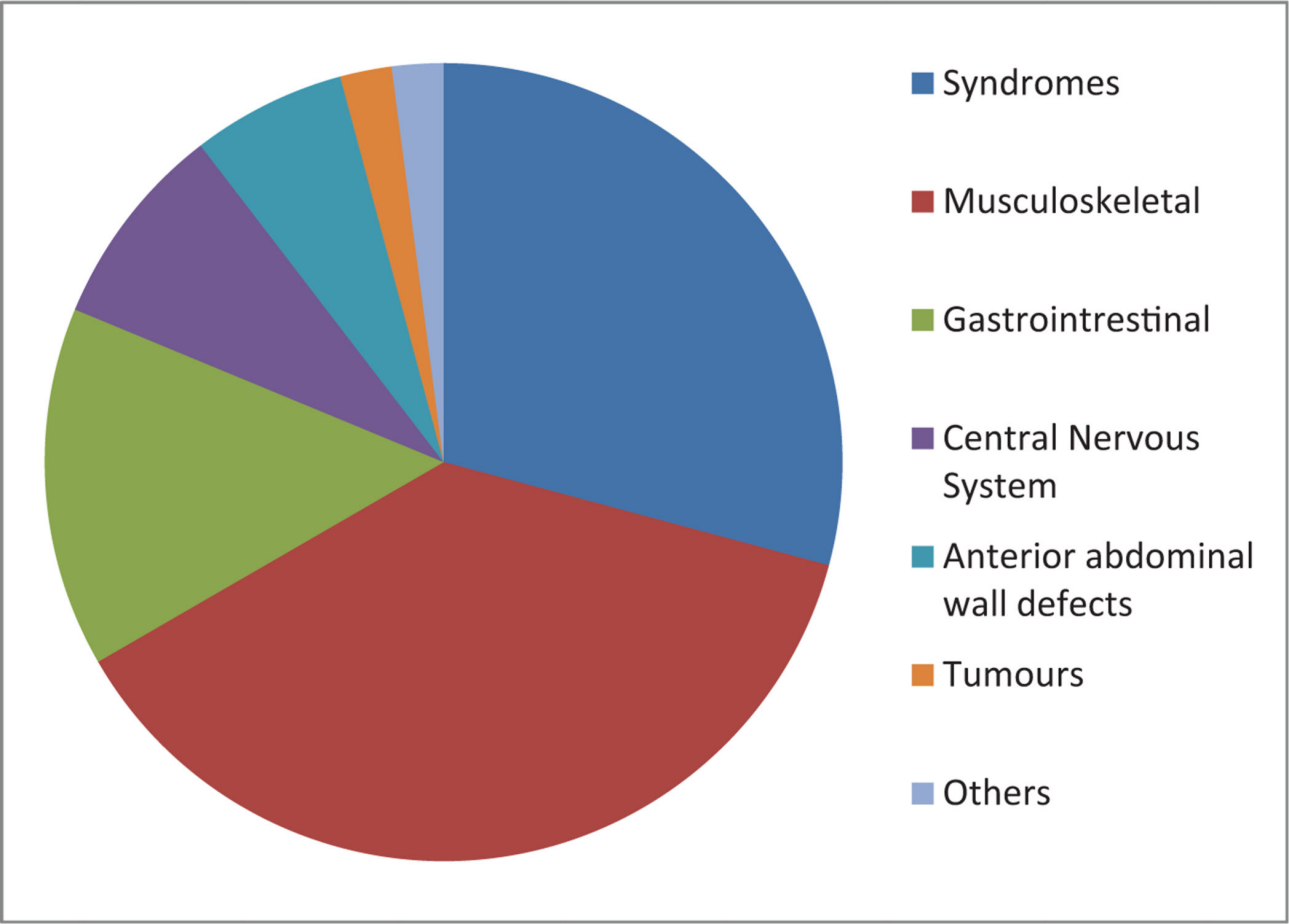
Classification of anomalies based on system affected.

Figure 1 shows the pattern of non- syndromic anomalies detected according to systems affected with the musculoskeletal system being the most common (36.7%). All neonates with an anomaly in the musculoskeletal system had polydactyly. Excluding the syndromes, the next frequent abnormality was the gastrointestinal in 14.3% (7/49), CNS in 8.2% (4/49) and anterior abdominal in 6.1% (3/49). Sacrococcygeal tumour and Prune Belly (others) were present in one neonate each.

CHD was detected in 15 neonates with external CA (prevalence of 306 per 1,000 or 30.6%) compared with 49 among neonates born without any congenital anomaly (prevalence 21.4 per 1,000) and this difference was significant statistically, *P* < 0.001.

CHD was more common in neonates with dysmorphic features and were detected in 78.6% (11/14) of the neonates with syndromes and 11.4% (4/35) of those with other external CA, *P*-value < 0.001.

Table 3 shows the pattern of CHD in neonates with CA. In neonates with Down syndrome, CHD was detected in 8/11 (72.7%), with the most common lesion being the coexistence of an atrial with a ventricular septal defect (ASD + VSD) in 37.5% (3/8) of them. Other CHD detected in these neonates included isolated VSDs and atrioventricular septal defects (AVSD) in two each, while one neonate had partial anomalous pulmonary venous drainage (PAPVR). All other three neonates with syndromes had a CHD; cor-triatriatum dexter was detected in the neonate with Barraister-Winter syndrome, a bicuspid aortic valve in the neonate with Turners’s syndrome and VSD in the trisomy 18.

In neonates with non-syndromic CA, CHD was detected in 28.6% (2/7) with anorectal malformations. One of the two neonates with spina bifida had ASD while only 5.6% (1/18) with polydactyly had a heart defect (ASD).

Hypertrophic cardiomyopathy (HOCM) was detected in 6.1% (3/49) including the neonate with Baraister-Winter syndrome whose mother had DM. The other two cases of HOCM were detected in 9.1% (1/11) with Down syndrome; who did not have a CHD and 33.3% (1/3) with omphalocoele major.

The factors associated with the presence of CHD in neonates with external CA are shown in Table 4. The mean age of the parents were significantly higher in the neonates with CHD; (32.2 ±6.6 years vs 28.6±6.6 years, *P* = 0.04) for the mothers and (40.6±7.8 years vs 36.0±6.4 years, *P* = 0.02) for the fathers. Neonates with external CA and CHD did not differ in relation to their weight, length, occipitofrontal circumference, gestational age and birth order when compared with those who did not have CHD [Table 1].

## DISCUSSION

In this study, we determined the prevalence of external CA in neonates born in two tertiary hospitals in Jos and explored their association with CHD. The prevalence of CA obtained in this study is similar to the 20.7 per 1,000 live births in the Niger- Delta region and the 22 per 1,000 in Anambra State.^[17,18]^ However, the previous studies included only major anomalies, both internal and external as opposed to this study were both minor and major external anomalies were studied.^[17,18]^ The prevalence of CA obtained in this study is however higher than the 5.3 per 1,000 obtained in the same region 15 years earlier which supports evidence that support changes over time.^[19]^ In Iran, the prevalence was shown to increase from 10.4 per 1,000 to 17 per 1,000 over a four year period.^[20]^

Compared to present study, a much lower prevalence of 6.5 per 1,000 was obtained in Edo state where external anomalies and CHD were included.^[21]^ In India, a pooled prevalence of 18.4 per 1,000 was obtained.^[22]^ These differences in prevalence have been shown to also vary with geographical location as there is interplay between genes and environmental factors in their aetiology.^[17]^

Excluding polydactyly which is a mild CA, the most common non- syndromic abnormality detected in this study were gastrointestinal (GIT) anomalies, a finding is similar to what was detected by Pam *et al.*^[19]^ in the same locality 15 years ago where neonates admitted were studied. Four cases of polydactyly were documented in this study but this was an incidental finding as the neonates were admitted primarily for neonatal sepsis.

The prevalence of CHD in babies with external CA is about 14 times higher than in normal babies. With the highest reported prevalence of CHD in the general population documented at 50 per 1,000 live births, it further supports the evidence that CHD is a lot more frequent in neonates with CA.^[23,24]^ Although no current studies on the prevalence of CHD in babies with external CA were found, it is vital that an echocardiography screening of these neonates is vital in determining associated co-morbidities that may worsen outcomes and alter clinical management. This is especially important in syndromic neonates who have CHD more commonly as detected also in present study, with reported frequencies of CHD ranging from 25% to 90% depending on the type of chromosomal abnormality present.^[10,24]^

Similar to other studies, Down syndrome was the most common type of dysmorphism, seen in about three- quarters of syndromic babies in this study.^[25]^ The occurrence of CHD among neonates with Down syndrome in this study is similar to the 74.1% obtained by Jalili et al in Iran but higher than the 40–50% reported by Cleves *et al.*^[26,27]^ Although some studies have reported that atrioventricular septal defects (AVSD) account for up to 40% of the CHDs detected in neonates with Down syndrome, only a quarter of the neonates in this study had this defect with ASD co-existing with VSD as the most frequent anomaly.^[28]^ Other studies have shown VSD to be more common than or as frequent as AVSD, a finding detected in present study.^[12,29]^ Tetralogy of Fallot, detected in one of the neonates with Down syndrome has also been documented as an observed CHD type in these children.^[24]^

In this study, the mean age of parents of children with external CA and CHD was significantly higher than the mean ages of those without CHD, in line with evidence suggesting that advanced age is associated with CHD.^[9]^ The present study was unable to show any association between maternal hypertension, diabetes mellitus and Human Immunodeficiency virus (HIV) infection and the presence of both CHD and external CA because of the small numbers of women with these abnormalities. Large databases from hospital and community- based surveillance systems will be useful in evaluating these associations.

Folic acid deficiency has been implicated as a risk factor in both spina bifida and CHD and it has been suggested that they may frequently co-exist.^[30]^ One of the two neonates with spina bifida in this study had ASD. Moeini et al however documented that CHD was not common in neonates with spina bifida.^[31]^ They found that only two out of 75 children with spina bifida had CHD. These children were female who also had skeletal anomalies implying that aetiology may not be necessarily linked to folic acid deficiency alone but could be multifactorial.^[32]^

Although hypertrophic cardiomyopathy (HOCM) is common in certain syndromes such as Noonan and Beckwidth-Weidemann,^[33]^ none of the neonates with HOCM in this study were clinically diagnosed with any of these syndromes. The neonate with HOCM and a clinical diagnosis of Baraister-Winter syndrome was born to a diabetic mother, another common risk factor for HOCM.^[34]^ Genetic studies that could have more accurately diagnosed these syndromes were beyond the scope of this study.

## CONCLUSION

We demonstrated that CA is common in Jos, Nigeria with a prevalence that is comparable to what is obtained in other parts of the world. CHD is more frequently found in babies with external CA especially in syndromic neonates and those with older parents. Hospital-based surveillance systems should be strengthened in order to provide current data and identify factors associated with the morbidity and mortality of individuals with CA in Nigeria.

## Figures and Tables

**Table 1:** Descriptive characteristics of neonates with congenital anomalies

Characteristics	Mean ± SD	Range

Gender		
Male − No (%)	27 (55.1)	-
Female − No (%)	22 (44.9)	-
Mean weight (kilograms)	3.1 ± 0.7	1.4–4.8
Mean length (centimeters)	48.5 ± 3.7	40–57
Occipitofrontal circumference (centimeters)	34.7 ± 2.0	29–39
Birth order	3.7 ± 2.3	1–12
Mean gestational age (weeks)	38.4 ± 1.8	34–42
Father’s age (years)	37.4 ± 7.1	23–60
Mother’s age (years)	29.8 ± 6.8	19–43
Previous child with congenital anomaly − No (%)	1 (2.0)	-
Previous miscarriage − No (%)	17 (34.7)	-
HIV positive mother − No (%)	2 (4.1)	-
Maternal hypertension − No (%)	5 (10.2)	-
Maternal diabetes mellitus − No (%)	1 (2.0)	-

**Table 2:** Pattern of congenital anomalies identified

Congenital anomalies	Total (*n*= 49)	Percentage	Prevalence per 1,000 live births	Male (*n*= 27)	Female (*n*= 22)

Syndromes	14	28.0	6.0	7	7
Down syndrome^a^	11	78.7	4.7	6	5
Trisomy 18	1	7.1	0.4	1	0
Turner syndrome	1	7.1	0.4	0	1
Baraister-Winter syndrome	1	7.1	0.4	0	1
**Other congenital anomalies**	**35**	**72.0**	**15.4**	**20**	**15**
Omphalocoele^b^	3	8.6	1.3	3	0
Prune Belly Syndrome	1	2.9	0.4	1	0
Anorectal malformation	7	20.0	3.0	4	3
Encephalocoele	2	5.7	0.9	2	0
Spina bifida	2	5.7	0.9	1	1
Sacrococcygeal teratoma	2	5.7	0.9	0	2
Polydactyly	18	51.4	6.4	9	9

a Also had cleft lip and palate.

b Also had polydactyly

**Table 3:** Pattern of CHD among neonates with external congenital anomalies

Congenital anomalies		CHD		
Syndromes	No	Type	No	Percentage with CHD

Down syndrome^a^	11	Isolated ventricular septal defect	2	72.7
		Atrioventricular septal defect	2	
		Atrial and ventricular septal defect	3	
		PAPVR	1	
Trisomy 18	1	VSD	1	100.0
Turner syndrome	1	Bicuspid aortic valve	1	100.0
Baraitser-Wintersyndrome^b^	1	Cor triatriatum dexter	1	100.0
**Other external congenital anomalies**	No	Type	No	Percentage with CHD
Anorectal malformation	7	Atrial with ventricular septal defect	2	28.6
Spina bifida	2	Atrial septal defect	1	50.0
Polydactyly	18	Atrial septal defect	1	7.1
**Total**	**49**		**15**	

a One neonate also had HOCM.

b Also had HOCM

**Table 4:** Factors associated with the presence and/or absence of CHD in neonates with congenital anomalies

Characteristics	Total	CHD present	CHD absent	*P*-value

Gender				
Male	28	9	18	
Female	22	6	16	0.40
Mean weight (kilograms)	3.1 ± 0.7	3.0±0.7	3.1±0.7	0.66
Mean length (centimeters)	48.5 ± 3.7	47.4±5.0	48.8±3.2	0.87
Occipitofrontal circumference (centimeters)	34.7 ± 2.0	34.4±1.5	34.8±2.1	0.74
Birth order	3.7± 2.3	4.2± 2.5	3.4± 2.2	0.14
Mean gestational age (weeks)	38.4 ± 1.8	38.2±1.8	38.5±1.8	0.68
Mother’s age (years)	29.8 ± 6.8	32.2±6.6	28.6±6.6	0.04*
Father’s age (years)	37.4 ± 7.1	40.6±7.8	36.0±6.4	0.02*
Previous miscarriage Yes	17	5	12	
No	32	10	22	0.89

* Statistically significant
